# Proteomic and metabolomic analysis of GH deficiency-induced NAFLD in hypopituitarism: insights into oxidative stress

**DOI:** 10.3389/fendo.2024.1371444

**Published:** 2024-05-21

**Authors:** Yuwen Zhang, Peizhan Chen, Xuqian Fang

**Affiliations:** ^1^ Department of Endocrine and Metabolic Diseases, Shanghai Institute of Endocrine and Metabolic Diseases, Ruijin Hospital, Shanghai Jiao Tong University School of Medicine, Shanghai, China; ^2^ Shanghai National Clinical Research Center for metabolic Diseases, Key Laboratory for Endocrine and Metabolic Diseases of the National Health Commission of the PR China, Shanghai National Center for Translational Medicine, Ruijin Hospital, Shanghai Jiao Tong University School of Medicine, Shanghai, China; ^3^ Clinical Research Center, Ruijin Hospital, Shanghai Jiao Tong University School of Medicine, Shanghai, China; ^4^ Department of Pathology, Ruijin Hospital, Shanghai Jiao Tong University School of Medicine, Shanghai, China

**Keywords:** GH deficiency, NAFLD, fatty acid oxidation, NADPH regeneration, oxidative stress

## Abstract

**Objective:**

Individuals with hypopituitarism (HPs) have an increased risk of developing non-alcoholic fatty liver disease (NAFLD)/non-alcoholic steatohepatitis (NASH) due to growth hormone deficiency (GHD). We aimed to investigate the possible mechanisms underlying the relationship between GHD and NAFLD using proteomic and metabolomic insights.

**Methods:**

Serum metabolic alternations were assessed in male HPs using untargeted metabolomics. A rat model of HP was established through hypophysectomy, followed by recombinant human growth hormone (rhGH) intervention. The mechanisms underlying GHD-mediated NAFLD were elucidated through the application of label-free proteomics and phosphorylation proteomics.

**Results:**

Metabolomic analysis revealed that biomarkers of mitochondrial dysfunction and oxidative stress, such as alanine, lactate, and creatine, were significantly elevated in HPs compared to age-matched controls. In rats, hypophysectomy led to marked hepatic steatosis, lipid peroxidation, and reduced glutathione (GSH), which were subsequently modulated by rhGH replacement. Proteomic analysis identified cytochrome P450s, mitochondrial translation elongation, and PPARA activating genes as the major distinguishing pathways in hypophysectomized rats. The processes of fatty acid transport, synthesis, oxidation, and NADP metabolism were tightly described. An enhanced regulation of peroxisome β-oxidation and ω-oxidation, together with a decreased NADPH regeneration, may exacerbate oxidative stress. Phosphoproteome data showed downregulation of JAK2-STAT5B and upregulation of mTOR signaling pathway.

**Conclusions:**

This study identified proteo-metabolomic signatures associated with the development of NAFLD in pituitary GHD. Evidence was found of oxidative stress imbalance resulting from abnormal fatty acid oxidation and NADPH regeneration, highlighting the role of GH deficiency in the development of NAFLD.

## Introduction

Hypopituitarism presents with a metabolic syndrome, characterized by decreased muscle mass, abnormal lipid metabolism, increased visceral fat deposition, and increased mortality, primarily due to an increased risk of cardiovascular disease (1). HPs exhibit an increased prevalence of NAFLD/NASH (2, 3). It has been reported that childhood-onset HPs, irrespective of its etiology, has an increased likelihood of progressing to NASH with advanced fibrosis or cirrhosis (4). In adults with HPs, liver dysfunction and hyperlipidemia are frequently observed (5, 6).

NAFLD is a significant complication observed in HPs, mainly due to growth hormone deficiency (GHD) (7). Although other factors such as thyroid hormone deficiency (8), and low serum testosterone levels (9) have also been indicated has potential associations with NAFLD. Considering that GH secretion is most commonly impaired in HPs, GH replacement therapy has been demonstrated to improve elevated levels of liver enzyme, reduce hepatic steatosis and histological changes, while also leading to a decrease in fibrotic markers (10, 11). Thus, indicating that GHD plays a dominant role in the development of NAFLD/NASH in these patients.

The role of the GH/IGF-1 axis in the pathogenesis of NAFLD/NASH is influenced by various metabolic, genetic, and microbiome-related factors (12). The association between serum levels of GH, IGF-1, and IGF-binding protein 3 (IGF-BP3) and hepatic steatosis and fibrosis in patients with NAFLD has been reported, even among individuals without GH deficiency (13). The GHR-JAK2-STAT5 signal pathway plays an important role in the pathogenesis of NAFLD/NASH. The development of hepatic steatosis has been observed in mice with liver-specific deletion of the GH receptor (GHR) (14, 15), liver-specific JAK2-deficient mice (16), or STAT5-deficient mice (17).

Kymberly D. Watt summarized the relationship of GHR-JAK2-STAT5 signal and NAFLD, mainly attributed to *de novo* lipogenesis and insulin resistance (18, 19). A reduction in GHR signaling results in the upregulation of lipogenesis genes, including peroxisome proliferator-activated receptor gamma (PPAR-γ), downstream CD36, lipoprotein lipase (LPL), and very low-density lipoprotein receptor (VLDLR). This subsequently increases lipogenesis, triglyceride accumulation, ultimately leading to hepatic steatosis.

In addition to the direct function of GHR signals, the downstream target IGF-1 plays an essential role in fatty liver (20, 21). In the hepatocytes of GH-deficient rats, mitochondrial morphology was severely impaired, and IGF-1 reversed these changes in the mitochondria (20). The administration of IGF-1 resulted in a reduction in oxidative mitochondrial damage, an improvement in complex V ATPase activity, and a decrease in caspase activities (22). The administration of IGF-1 has also been reported to enhance liver dysfunction and fibrosis, as well as improve mitochondrial function in aging rats (23). Collectively, the administration of IGF-1 enhances insulin sensitivity, reduces reactive oxygen species (ROS) levels, improves mitochondrial function, and attenuates triglyceride accumulation in hepatocytes.

Metabolomics and proteomics analysis may provide a novel approach to elucidate the pathogenesis of NAFLD. Matej Orešič et al. developed a metabolomic approach to identify circulating lipid signatures associated with liver fat content and NASH, and found oxidative stress-buffering potential via ether lipids in late-stage NASH (24). Potential serum metabolites as markers of NAFLD was found in individuals with early-stage NAFLD (25). Anstee et al. presented a proteo-transcriptomic map that elucidates the molecular changes occurring during the progression of NAFLD, to enhance understanding the disease’s pathogenesis and for identifying novel therapeutic targets (26). In this study, metabolic changes in patients with HP were identified, and the focus was mainly on exploring the differential metabolites associated with mitochondrial function and oxidative stress. Subsequently, a proteomics analysis was performed to gain insights into the molecular mechanisms underlying GHD-mediated NAFLD in hypophysectomized rats, both before and after rhGH intervention.

## Methods

### Participant recruitment

Patients with health controls were randomly recruited at Ruijin Hospital (Shanghai, China) between January 2016 and December 2018. Recruitment for the current study followed the same protocol as our previously published study (27). The study was conducted on 134 patients diagnosed with HPs (**Supplementary Table 1**), including congenital (n = 83) and acquired (n = 51). Congenital pituitary HPs stems from pituitary stalk interruption syndrome, while acquired HPs results from surgical or radiation injury during childhood. The diagnosis of GHD was defined as a peak GH level ≤5 μg/L on an insulin tolerance test. The exclusion criteria were as follows (1): Evidence of viral, inherited autoimmune, cholesteric, or drug-induced liver disease (2); excessive alcohol consumption (>210 g/week in men) (3); Incomplete data. There are 90 healthy controls recruited with matched gender, BMI, and exclude NAFLD by liver ultrasound. The study protocol received approval from the Board of Medical Ethics at Ruijin Hospital, Shanghai Jiao Tong University School of Medicine, China. All patients provided informed consent to participate in the study.

### Hormone replacement plan of HPs

To control for potential confounding variables, 133 male patients with GHD were enrolled. After diagnosis (median diagnosis age of 16.50 years), physiologic dosages of glucocorticoids and/or thyroid hormone were administered. Some patients received GH therapy during childhood and discontinued for at least 24 months. Gonadotropin treatment was administered to patients with LH/FSH deficiency for at least 24 months.

### Quantitative determination of metabolites by untargeted metabolomics

Analyses were performed using a UHPLC system (1290 Infinity LC, Agilent Technologies) coupled to a quadrupole time-of-flight instrument (AB SCIEX, California, USA). The stability and repeatability of the instrument analysis were monitored using quality control (QC) samples. In brief, 80 μL of an ethylene diamine tetra acetic acid serum sample was deproteinized with 1 mL of methanol and subsequently purified through ion exchange columns as previously described (28). Variable importance in projection (VIP) values for each variable in the partial least squares-discriminant analysis (PLS-DA) model were calculated to identify metabolites contributing to classification. Variables with *P*-values < 0.05 and VIP values > 1 were considered statistically significant.

### The hypophysectomized rat models

30 Sprague-Dawley rats, aged 3-4 weeks and weighing 70-80 g, were randomly divided into three groups. Two groups underwent hypophysectomy using the methods described by Honeyman et al. (19). After 2 weeks’ recovery, one of the hypophysectomized groups was administered rhGH (0.1 mg/kg/day) via subcutaneous injection for 2 weeks. All rats were housed in an environment with a room temperature of 20–22°C, alternating 12-h periods of light and dark, and free access to a standard chow diet. Body weight and fasting blood samples were collected before and two weeks after the operation, and two weeks after rhGH intervention. At the end of the study, the overnight-fasted rats were euthanized. The livers were excised and frozen immediately in liquid nitrogen. Blood samples were collected, and plasma was obtained by centrifugation (2200× g, 4°C) and stored at −80°C.

### Liver histology

Liver samples were fixed in 10% phosphate-buffered formalin and subsequently embedded in paraffin. Microtome sections with a thickness of five microns were obtained. Hematoxylin and eosin (H&E) staining was performed on the sections, and they were examined by a pathologist specializing in liver tumors at Ruijin Hospital. Oil Red O staining was carried out using a commercial kit (C0157M; Beyotime, China).

### Determination of lipid peroxidation MDA and glutathione GSH

Liver samples were thoroughly rinsed in ice-cold PBS (0.01M, pH=7.4) to remove excess blood. Tissue pieces were weighed, minced into small pieces, and homogenized in PBS. Malondialdehyde (MDA) is a natural product of lipid oxidation in living organisms, which was used to detect the level of lipid oxidation. The concentration of MDA in serum and liver tissue was determined using a Malondialdehyde (MDA) detection kit (thiobarbituric acid reactive substances (TBARS) assay, mlbio, China). Glutathione (GSH) concentrations were detected by an enzymatic assay using the Rat GSH ELISA Kit (ELISA method, mlbio, China). The concentration of GSH in the samples was determined by comparing the O.D. of the samples to the standard curve, following the detailed operational steps outlined in the instruction manual.

### 4D label free relative quantitative proteome and phosphorylation proteomics

Liver proteins were extracted and lyzed in SDT buffer (4% SDS, 100 mM Tris-HCl, 1 mM DTT, pH 7.6). The protein concentration was quantified using the BCA Protein Assay Kit (Beyotime Biotechnology, CN). For filter-aided sample preparation, 200 μg of protein was mixed with 30 μL of SDT buffer. Phosphopeptides were enriched using the High-SelectTM Fe-NTA Phosphopeptides Enrichment Kit (Thermo Scientific, CN). Ten cycles of PASEF MS/MS were performed with a target intensity of 1.5 k and a threshold of 2500, leading to 4D label-free phosphorylation proteomics being conducted at Applied Protein Technology Co., Ltd.

Each group had three or four repetitions, and the criteria used were based on fold change (FC) (greater than 2.0-fold up-regulation or less than 0.5-fold down-regulation) and a *P*-value of < 0.05, which were used to determine the count of up-regulated and down-regulated proteins among the comparison groups.

### Statistical analysis

The Kolmogorov–Smirnov statistical tests was performed to assess data normality. Continuous variables were presented as the mean ± SD for normally distributed variables or medians (interquartile ranges) for the skewed variables. The group differences were compared using Student’s t-test or MannWhitney U-test. All analyses were performed with SPSS software version 23.0 (SPSS Inc., Chicago, IL, United States). Significance tests were two-tailed, and statistical significance was set at *P* < 0.05.

### Bioinformatics analysis

PCA was carried out using online analysis software (https://www.omicsolution.org/wkomics/main/). The PANTHER software was used for the functional category of Gene Ontology (GO) (http://pantherdb.org/). Pathways associated with differential protein expression were identified using the Kyoto Encyclopedia of Genes and Genomes (KEGG) (http://www.kegg.jp/kegg/).

## Results

### Serum biomarkers of mitochondrial dysfunction and oxidative stress

**Figure 1:**
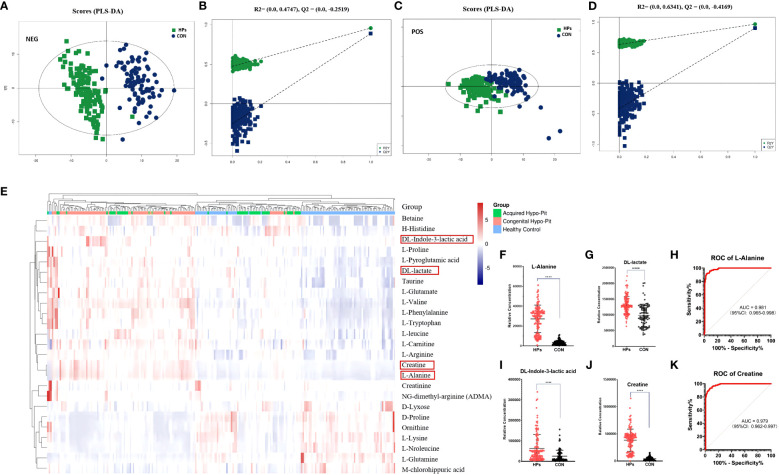
Serum biomarkers of mitochondrial dysfunction and oxidative stress were elevated in patients diagnosed with hypopituitarism. PLS-DA plot of hypopituitarism vs. healthy controls in negative ion **(A, B)** and positive ion **(C, D)**. Circulated amino acids between hypopituitarism and health controls **(E)**. Patients diagnosed with hypopituitarism exhibited higher serum levels of alanine **(F)**, DL-lactic acid **(G)**, DL-indole-3-lactic acid **(I)**, and creatine **(J)**. The AUC of alanine **(H)** and creatine **(K)** was 0.981 and 0.979, respectively, for distinguishing hypopituitarism from healthy controls. ****P < 0.0001.

Metabolomics analysis identified serum biomarkers of mitochondrial dysfunction and oxidative stress in patients with HP. The supervised PLS-DA plots identified distinct metabolic profiles between HPs and CTs for both positive and negative modes (**Figures 1A–D**). A total of 72 annotated metabolites with VIP > 1 and *P* < 0.05 were identified between the HPs and CTs (**Supplementary Table 2**). Among these, 25 (34.7%) metabolites belonged to the subclass of amino acids, 17 to fatty acids, 12 to glycerin phospholipid, 4 to steroids, 3 to nucleic acid, and 2 to bile acids, with the rest falling into other categories.

The heatmap for differential abundant amino acids was shown in **Figure 1E**. Patients with PH had higher levels of alanine, phenylalanine, arginine, glutamate, pyroglutamic acid, leucine, valine, histidine, proline, and tryptophan, while the serum concentrations of glutamine, lysine, and norleucine was significantly decreased (all P < 0.001). Particularly, patients with HPs had higher levels of alanine (7.96-fold), DL-lactic acid (1.33-fold), DL-indole-3-lactic acid (2.49-fold), and creatine (9.28-fold) (**Figures 1F, G, I, J**). Among these markers, alanine and creatine were observed to be reliable metabolic markers in HPs, with diagnostic areas under the curve of 0.981 (95% CI: 0.965-0.998) and 0.979 (95% CI: 0.962-0.997), respectively (**Figures 1H, K**).

### Hepatic steatosis, lipid peroxidation and oxidative stress in hypophysectomized rats

**Figure 2:**
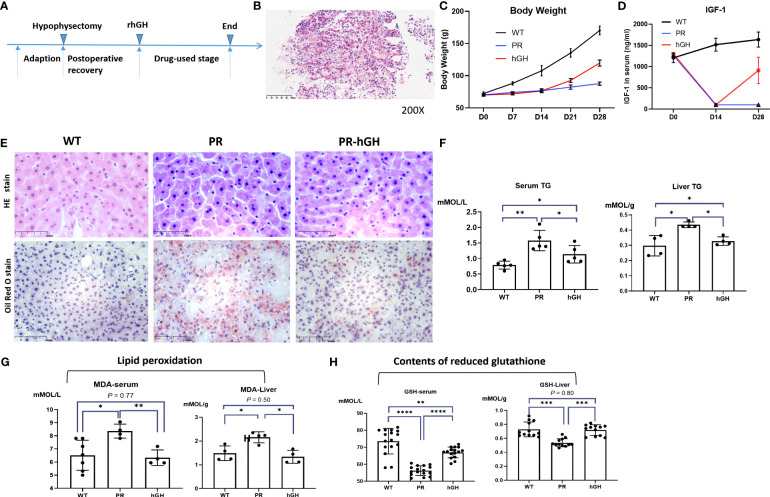
Establishment of the hypophysectomized rat model and hepatic pathology changes. **(A)** Timeline for constructing the animal model; **(B)** Confirmation of pituitary localization through frozen pathology; Weight changes **(C)**, IGF-1 levels **(D)** during the construction process of the animal model; **(E)** Hepatic pathology in the control group (WT), PR group, and PR-hGH group revealed by H&E and Oil Red O staining; **(F)** The concentration of TG both in serum and liver; **(G)** Lipid peroxidation levels were assessed in both liver and serum samples; **(H)** The reduced glutathione levels were determined using ELISA in both liver and serum samples. PR group-pituitary resection group; PR-hGH group-rhGH intervention after pituitary resection group; WT group-wild type group. *P < 0.05, **P < 0.01, ***P < 0.001 , ****P < 0.0001.

After hypophysectomy, rats experienced severe loss of appetite and significant weight loss. However, for two weeks rhGH intervention, they gained weight significantly (**Figure 2**). To confirm proper extraction of the pituitary gland, serum IGF-1 levels were consecutively assessed. The IGF-1 level was decreased by more than 80% in hypophysectomized rats (PR group), and it significantly increased after rhGH intervention (PR-hGH group). H&E and Oil Red O Staining revealed hepatic steatosis in these rats, along with an increase in triglyceride (TG) content in serum and liver, which is similar to mouse models of steatosis resulting from the knockout of hepatic GHR (15, 29). In addition to abnormal lipid metabolism, the hypophysectomized rats had notably increased lipid peroxidation and decreased reduced glutathione (GSH). These abnormalities were subsequently modulated by rhGH intervention both in serum and liver tissue.

### Overview of DEPs in hypophysectomized rats by proteomics analysis

**Figure 3:**
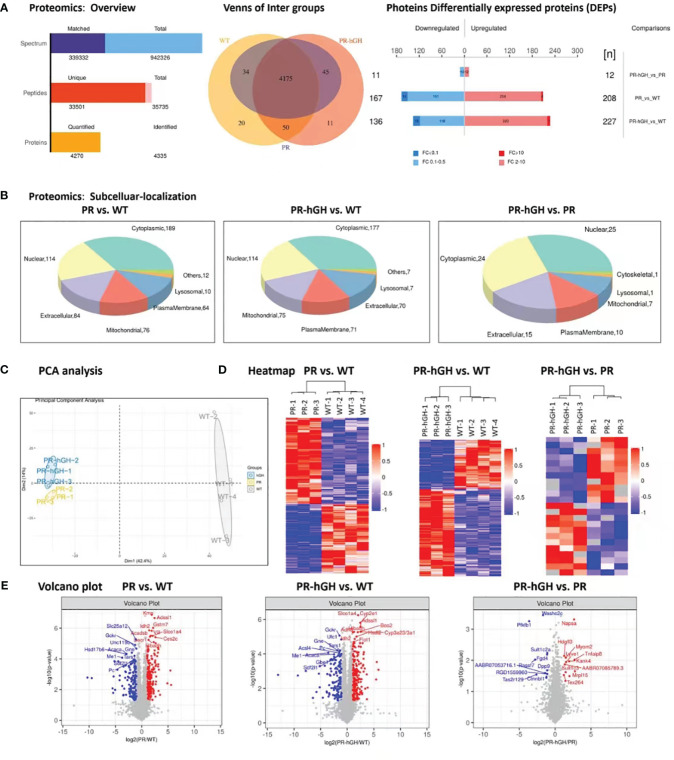
Proteome differences between liver samples from PR, PR-hGH and WT groups. **(A)**: Overview of differentially abundant proteins; **(B)**: Subcellular localization of the differentially abundant proteins; **(C)**: Principal component analysis (PCA) clearly separates proteomes from PR, PR-hGH and WT; **(D)**: Heat map of the differentially abundant proteins; **(E)**: Volcano plot of log_10_ fold changes of DEPs. Top10 differentially abundant proteins are highlighted. Note: PR group-pituitary resection group; PR-hGH group-rhGH intervention after pituitary resection group; WT group-wild type group.

In overall of proteomics (**Figure 3A**), a total of 4175 protein groups were detected and used for further analyses in all groups. The statistical analysis of the proteome data set, conducted using a two-way ANOVA, revealed that 208 and 167 protein groups were upregulated and downregulated, respectively, in PR compared to WT. In PR-hGH, 363 protein groups were dysregulated compared to WT, while only 23 were dysregulated between PR and PR-hGH. In subcellular localization analysis (**Figure 3B**), the DEPs mainly located in cytoplasmic, nuclear, extracellular, and mitochondrial regions.

As demonstrated in the PCA and heatmap analyses (**Figures 3C,D**), the PR and WT groups, as well as the hGH and WT groups, can be distinctly separated, whereas the PR and hGH groups are relatively close to each other. They exhibit improved separation in the OPLS-DA analysis, with a predictability of 0.31 and a model interpretation rate of 0.99 (**Supplementary Figure 1**). Among the most significantly downregulated proteins after hypophysectomy were GCKR, SLC25A12, ME1, and PC, while the significantly upregulated proteins included KMO, IDH2, Adssl1, Acadsb, Vill, Decr1, and others. These proteins are involved in fatty acid metabolism and the fatty acids oxidation process. Besides, ME1, IDH2, GCKR, and PC were the key proteins that contributed to carbohydrate and glucose metabolic processes. On the other hand, the most significantly regulated proteins between PR-hGH and PR were NAPSA, PFKFB1, SULT1C2A, SULT1C3, and HDGFL3 (**Figure 3E**).

### Functional enrichment of differentially abundant proteins

**Figure 4:**
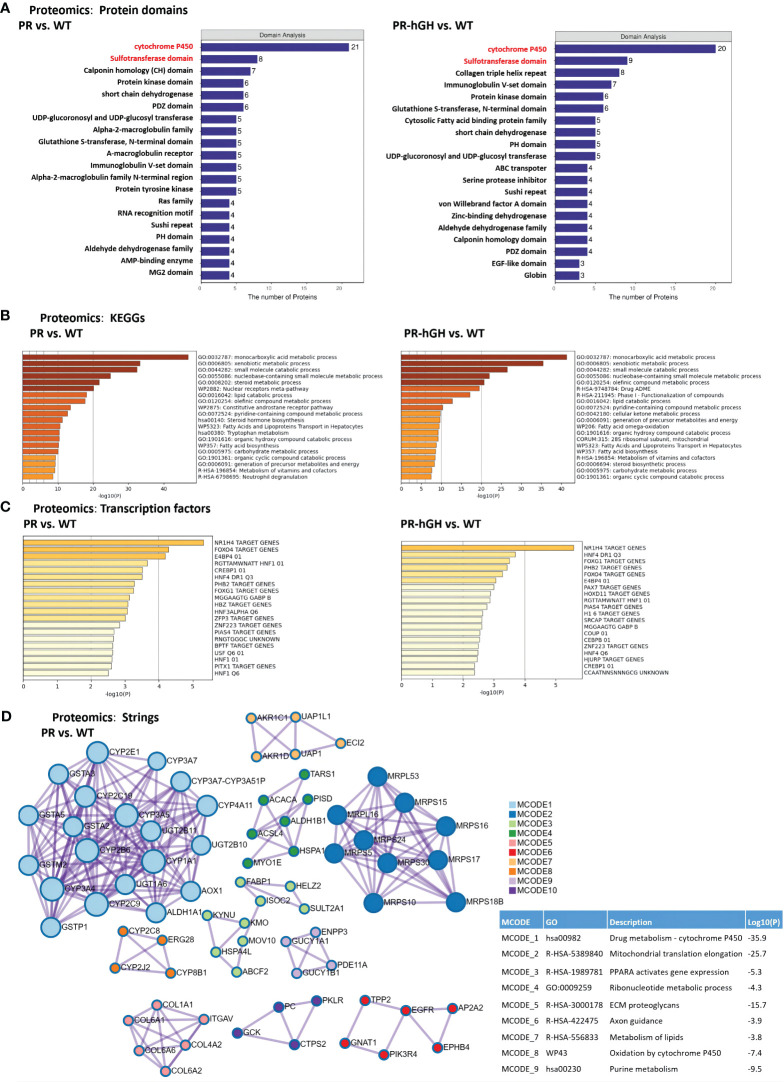
Functional enrichment of DEPs between PR and WT, PR-hGH and WT. **(A)** Enrichment by protein domains; **(B)** ClueGO functional enrichment analysis for proteins significantly dysregulated; **(C)** Enrichment by transcription factors; **(D)** Protein-protein interactions among DEPs, the analysis used the term GO Biological Process and KEGG pathways. PR group-pituitary resection group; PR-hGH group-rhGH intervention after pituitary resection group; WT group-wild type group.

Phase I metabolic enzyme cyp450s and the phase II metabolic enzyme sulfate transferases hit major DEPs by protein domains between PR and WT, and similar results were observed between PR-hGH and WT (**Figure 4A**). KEGG pathway enrichment analysis indicated that the pathways with the largest number of DEPs were xenobiotic metabolic process, steroid metabolic process, lipid catabolic process, and carbohydrate metabolic process (**Figure 4B**).

Cytochrome P450s-associated proteins were involved in xenobiotic metabolic process, steroid hormone biosynthesis, bile acid metabolism, and long-chain fatty acid metabolism. Twenty-one cytochrome P450 enzymes were significantly dysregulated in the PR liver samples. Specifically, CYP2J4, CYP2E1, CYP3A23/3A1, CYP3A18, CYP2B2, CYP2C6V1, CYP3A62, CYP4A1, CYP2C6V1, CYP8B1, CYP2A1, CYP4A3, and CYP2C23 were upregulated, while CYP7A1, CYP2T1, CYP2B3, CYP2J10, CYP2C11, CYP2C7A, and CYP2C12 were downregulated. Among them, the expression of CYP2E1, CYP4A1, and CYP4A3 was significantly upregulated in PR. CYP2E1 and CYP4A are inducible hepatic microsomal cytochrome P450s involved in the hydroxylation of fatty acids and can initiate the process of lipid peroxidation (30).

Transcription factor target enrichment revealed 15 NR1H4 target proteins, also known as bile acid receptors, which were identified among the dysregulated proteins. These proteins included AGT, AGXT, CYP2E1, GCKr, ICAM1, KNG1, PFKFB1, SERPINA1, POR, SELENBP1, TOM1, CSAD, ETNK2, VKORC1, and NTAN1 (**Figure 4C**). Protein-protein interactions among the dysregulated proteins showed interactions with CYP450s, mitochondrial translation elongation, PPARA activation of gene expression, ribonucleotide metabolism, and ECM proteoglycans (**Figure 4D**).

### Differentially expressed proteins in lipid metabolism

**Figure 5:**
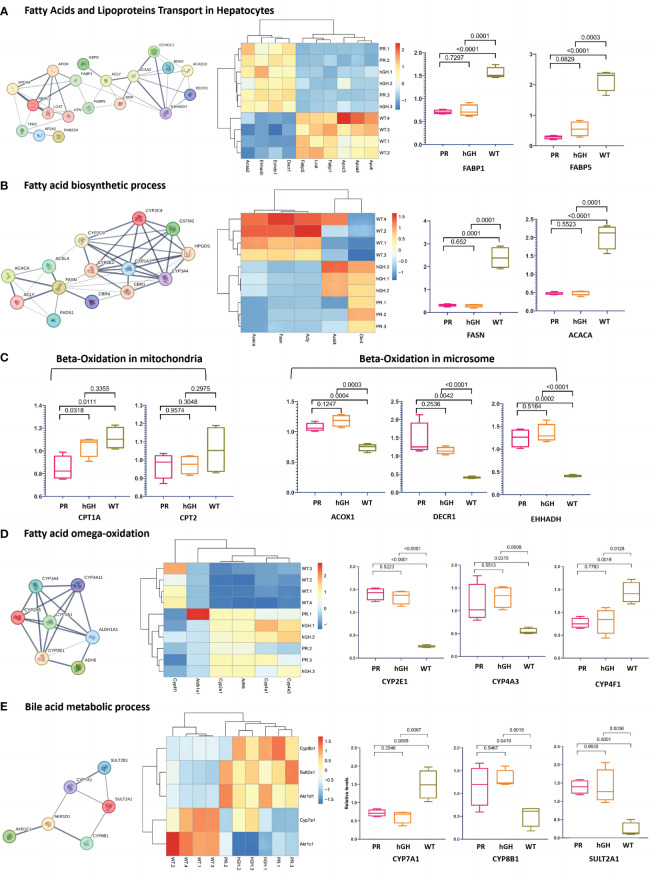
Differentially abundant proteins in lipid metabolism. **(A)** Protein-protein interactions in fatty acids and lipoproteins transport in hepatocytes; **(B)** Protein-protein interactions in fatty acid biosynthetic process; **(C)** Main DEPs in fatty acid beta-oxidation; **(D)** Main DEPs in fatty acid omega-oxidation; **(E)** Protein-protein interactions in bile acid metabolic process. PR-pituitary resection group; PR-hGH-rhGH intervention after pituitary resection group; WT-wild type group.

In the gene set of “Fatty acids and lipoprotein transport” (**Figure 5A**), there was a significant decrease in FABP1, FABP5, APOC3, APOA4, APOH, and LCAT, while the abundance of ACAA2, ECHDC1, EHHADH, and DECR1 increased. In the gene set of “Fatty acid biosynthesis” (**Figure 5B**), there was a decrease in FASN, ACACA, and ACSL4, while there was an increase in CBR4 and ACLY in PR. Surprisingly, FASN (FAS) and ACACA (ACC1), which are rate-limiting enzymes in *de novo* lipogenesis, decreased in hypophysectomized rats. Notably, ACSL4 was the only protein that showed a significant reversal after rhGH intervention.

In the gene set of “Fatty acid oxidation” (**Figure 5C**), hypophysectomized rats showed a reduced β-oxidation in mitochondria and a compensatory increase in microsomal oxidation. CPT1A, which plays a key role in transporting long-chain fatty acids into mitochondria for oxidation, exhibited a decrease in PR with a fold change of 0.76 compared to WT. However, no significant difference was observed in CPT2. The degradation of long-chain and unsaturated fatty acids is primarily carried out by ACOX1, CAT1, DECR1, and EHHADH through fatty acid β-oxidation in peroxisomes and ω-oxidation in the endoplasmic reticulum and peroxisomes (31). Our findings revealed that ACOX1, DECR1, and EHHADH increased significantly, with fold changes of 1.45, 3.54, and 3.05, respectively, compared to WT. This suggests that microsomal oxidation may serve as an alternative pathway when mitochondrial function is dysregulated in GHD states. CYP4As and CYP4F are enzymes involved in ω-oxidation of fatty acids through hydroxylation at the ω-carbon position. Proteomics data showed an increased expression of CYP4As but a decreased expression of CYP4F in PR and PR-hGH (**Figure 5D**).

In gene set of “Bile acid metabolic process” (**Figure 5E**), Cyp7a1 and Akt1c1 decreased, while Cyp8b1, Sult2a1 and Akr1d1 increased in PR and PR-hGH.

### Differentially expressed proteins in NADP metabolic process

**Figure 6:**
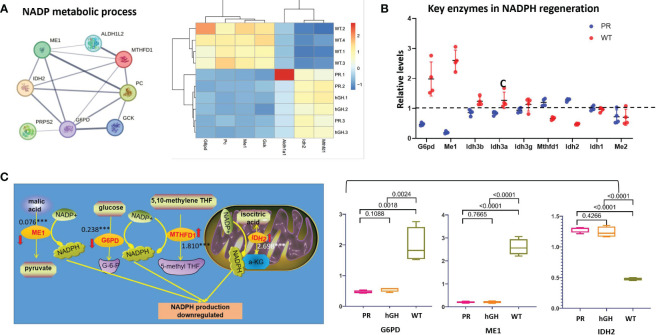
Differentially expressed proteins in NADP metabolic process. **(A)** Protein-protein interactions in NADP metabolic process; **(B)** Key enzymes in NADPH regeneration; **(C)** Changes of main NADPH regeneration pathways. PR group-pituitary resection group; PR-hGH group-rhGH intervention after pituitary resection group; WT group-wild type group.

In the gene set of “NADP metabolic process” (**Figure 6A**), G6PD, PC, ME1, and GCK decreased, while ALDH1A1, IDH2, and MTHFD1 increased in PR. We examined the expression levels of critical enzymes in key pathways responsible for NADPH regeneration, including glucose-6-phosphate dehydrogenase (G6PD) in the pentose phosphate pathway (PPP), malic enzymes (ME1 and ME2) in the malic enzyme pathway, isocitrate dehydrogenases (IDH1-IDH3) in the isocitrate dehydrogenase pathway, and MTHFD1 and MTHFD2 in the folic acid pathway. Among these enzymes, G6PD, ME1, MTHFD1, and IDH2 showed significant differences between PR and WT (**Figure 6B**).

In detail, the levels of ME1 and G6PD notably decreased after hypophysectomy, reaching 0.075 and 0.237 times lower than those of the control group, respectively (**Figure 6C**). The content of ME1 and G6PD slightly restored after rhGH intervention, but no significant difference occurred. The expression of MTHFD1 and IDH2 increased 1.810-fold and 2.697-fold in PR respectively, compared to WT. In summary, the results showed that the protein levels of ME1 and G6PD were significantly reduced, while MTHFD1 and IDH2 exhibited compensatory increases.

### Overview of phosphorylation proteomics

There are 334 up-regulated phosphor-proteins (DEPPs) and 585 down-regulated DEPPs between PR-hGH and PR (**Supplementary Figure 2**). A two-dimensional principal component analysis (2D-PCA) and heat map analysis could clearly separate PR, PR-hGH, and WT. As shown in the volcano plot between PR-hGH and PR, the most significantly downregulated proteins included ISCA2, DCK, LPIN1, KSE1, ACTB, and ALDH9A1, while ALDOB, TRIM28, UST4R, TRA2A, CAPZB, UGDH, and CPS1 were upregulated.

### Functional enrichment of differentially abundant phosphorylated proteins

Both KEGG and Protein-protein interactions revealed “diseases of signal transduction by growth factor receptors and second messengers”, “metabolism of lipids”, and “amino acid metabolism” significantly disturbed between PR-hGH and PR (**Supplementary Figure 3**).

### Jak2-Stat5B and mTOR signal pathway

**Figure 7:**
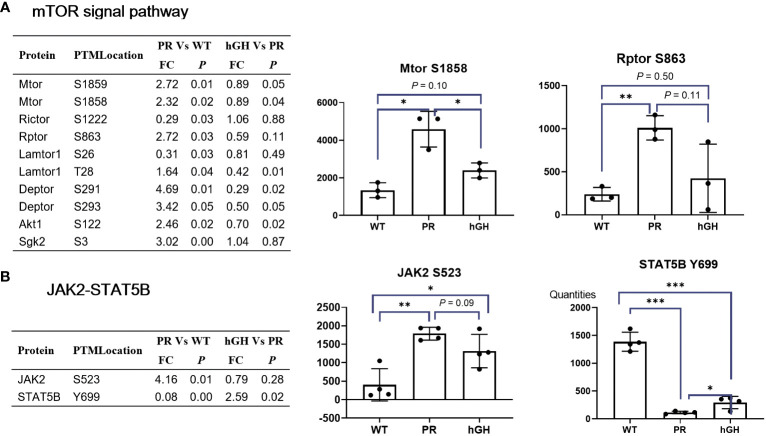
mTOR and Jak2-Stat5B signal pathway. **(A)** Differentially abundant phospho-proteins in mTOR signal pathway. **(B)** Differentially abundant phospho-proteins in JAK2-STAT5B signal pathway. PR group-pituitary resection group; PR-hGH group-rhGH intervention after pituitary resection group; WT group-wild type group. *P < 0.05, **P < 0.01, ***P < 0.001.

Gene set enrichment analysis (GSEA) revealed that mTORC1-related signatures enriched in hypophysectomized rats (**Figure 7**). The phosphorylation of MTOR S1858/S1859, RPTOR S863, Lamtor1 T28, and DEPTOR S291/S293 was increased. The Rptor (an mTOR-interacting partner) S863 is a master biochemical switch that modulates hierarchical raptor phosphorylation, which regulate mTORC1 (32). The known substrate and feedback regulation of mTORC1, phosphorylation of Akt1 S122 and Sgk2 S3 were also increased. After rhGH intervention, most phosphorylation sites in the mTOR signal pathway partially restored, and MTOR S1858/S1859, Lamtor1 T28, DEPTOR S291/S293, and Akt1 S122 reached significant differences between PR-hGH and PR. In the JAK-STAT signaling pathway, JAK2 S523 increased by 4.16-fold changes after hypophysectomy, while STAT5B Y699 dephosphorylated markedly in PR, but partially restored after two weeks of rhGH intervention.

## Discussion

In metabolomics analysis, a large proposition (17/25) of amino acids is significantly increased in HP, indicating an elevated degradation of amino acids, similar to the findings in a growth hormone receptor-deficient pig model (33). Among these altered amino acids, alanine, lactate, and creatine exhibited significant increases in serum of HPs compared to age and BMI-matched controls. High blood levels of alanine and lactate may serve as indicators of oxidative stress, as they can be generated from ROS in the liver (34, 35). Creatine has been identified as a potential biomarker of human mitochondrial dysfunction with clinical utility (36). Mitochondrion is a key organelle in cellular bioenergetics. Mitochondrial dysfunction can stem from both inherited disorders and secondary mitochondrial dysfunction-related diseases, such as type 2 diabetes, obesity, and neurodegenerative diseases (37). Creatine serves as a universal energy currency for cell metabolism and is partially synthesized in mitochondria. In cases of mitochondrial dysfunction, disturbances in creatine synthesis or utilization may occur, leading to creatine release into the bloodstream (38). In studies of respiratory chain diseases, creatine has consistently shown elevated levels in two independent cohorts, surpassing lactate and alanine in the magnitude of elevation and statistical significance (36). Therefore, the elevated levels of alanine, lactate, and creatine suggest mitochondrial dysfunction and oxidative stress, which are closely associated with the development of NAFLD.

Dysregulated fatty acid metabolism plays a critical role in the pathogenesis of GHD-induced NAFLD. We observed the enzymes in microsome, which catalyze medium/long-chain fatty acids and arachidonic acid, were increased after hypophysectomy. Although mitochondrial β-oxidation plays a dominant role in fatty acid utilization, peroxisomal β-oxidation and microsomal ω-oxidation of specific FAs could also act an alternative way, particularly in cases of fatty acid oxidation disorders (39), conditions of starvation and diabetes (40). However, excessive microsomal oxidation in the liver can have detrimental effects (41). The breakdown of fatty acids through ω-oxidation generates ROS, H2O2 as byproducts, leading to oxidative stress and the initiation of lipid peroxidation (31). Cyp4A and Cyp4F are key enzymes in ω-oxidation. The expression panel of an increased CYP4A, but a decreased CYP4F was proposed as a progression of steatosis to steatohepatitis (42). Consistently, a similar tendency was also observed in our hypophysectomized rat model.

CYP2E1 and CYP4A appear to play significant roles in the development of NAFLD/NASH by promoting the accumulation of lipid, inflammation, and fibrosis through the generation of ROS and NADPH-dependent microsomal lipid peroxidation (43). The upregulation of CYP2E1 and CYP4A has been consistently observed in both clinical and experimental cases of NASH, indicating the presence of oxidative stress and mitochondrial injury (44). The regulation of hepatic CYP2E1 and CYP4A expression involves several factors that have been associated with the pathogenesis of hepatic steatosis and NASH (45). The *in vitro* studies conducted in hepatocyte cell lines overexpressing CYP2E1 indicate potential associations between CYP2E1-dependent oxidative stress, GSH homeostasis, and mitochondrial damage that ultimately result in cellular apoptosis (46). CYP2E1-generated ROS can activate a variety of pro-inflammatory pathways, including nuclear factor kappa B (NF-κB), c-Jun N-terminal kinase (JNK), and toll-like receptor 4 (TLR4) signaling (47). In summary, increased microsomal oxidation may play an important role in the development of GHD-induced hepatic steatosis and steatohepatitis. This may occur through its participation in NADPH consumption, oxidative stress, and lipid peroxidation.

NADPH acts as a vital reducing agent in numerous cellular processes that aim to counteract oxidative stress and maintain cellular homeostasis. In its reduced form, glutathione acts as a powerful intracellular antioxidant, protecting cells from the damaging effects of ROS. NADPH supplies the reducing equivalents required to convert oxidized glutathione (GSSG) back to its reduced form (GSH) (48). Consequently, deficiencies in NADPH production or utilization can lead to increased sensitivity to oxidative damage, contributing to the development of NASH, which is characterized by hepatic steatosis and inflammation.

One of major source of NADPH production is the PPP (49, 50). G6PD, rate-limiting enzyme of PPP, plays an importance role in cellular physiology as it is a major source of NADPH that is required by many essential cellular systems including the antioxidant pathways to fuel glutathione recycling, nitric oxide synthase, NADPH oxidase, cytochrome p450 system, and others (49). Several studies have linked the downregulation of G6PD expression with the development of NAFLD. For instance, a study in mice has shown that methionine-choline deficient induced NAFLD reduced G6PD expression and PPP activity in the liver, leading to increased oxidative stress and liver injury (51). Similarly, in a rat model of high fructose-induced steatohepatitis, nicotinamide mitigates liver steatosis and fibrosis by regulating redox homeostasis through a G6PD-dependent mechanism (52).

The PPP is a major source of NADPH production, but ME1 and isocitrate dehydrogenase 1 (IDH1) may provide alternative metabolic pathways to maintain NADPH homeostasis in conditions of glucose starvation (53). ME1 gene expression can be rapidly induced by insulin, thyroid hormone, PPAR ligands, and high-carbohydrate and high-fat diets (54). Several studies have shown that ME1 may be involved in adiposity and hepatic steatosis. For example, a study in rats with ME1 deficiency found that ME1 may promote adiposity and hepatic steatosis and induce circulating insulin and leptin, supporting the therapeutic targeting of ME1 for obesity, diabetes, and hepatic steatosis (55, 56).

In this study, we found both G6PD and ME1 dramatically decreased after hypophysectomy, while MTHFD1 and IDH2 significantly increased. The expression of MTHFD1 and IDH2 may represent some kind of compensation. When G6PD and ME1 are downregulated in cancer cells, adaptive upregulation of MTHFD1 (57), mitochondrial IDH2 (58), may serve to replenish the NADPH pool and mitigate cytosolic ROS. Thus, the adaptive upregulation of MTHFD1 and IDH2 may serve to replenish the NADPH pool.

After administering rhGH to hypophysectomized rats, the JAK2-STAT5B signaling pathway activated with evidence of restoration of circulating IGF-1 levels, growth promotion and metabolic effects of alleviation of fatty liver. JAK2, particularly the tyrosine residues Y1007 and Y1008 in the activation loop of its kinase domain, plays a critical role in its catalytic activity and regulation (59). However, our omics data did not detect any tyrosine residues in JAK2. Instead, we detected JAK2 Ser523 increased in PR group and partially restored in PR-hGH group. Recent studies have identified Ser523 as the first-described site of JAK2 serine phosphorylation, and it has been shown to inhibit JAK2-dependent leptin receptor signaling (60). Our data suggest that JAK2 Ser523 plays a suppressive role, as the JAK2-STAT5B signaling pathway was activated after rhGH intervention. STAT5B dimerization and nuclear translocation, which regulate the transcription of target genes involved in various biological processes, depend on the phosphorylation of STAT5B Tyrosine 699 (Y699). Our data showed a decrease in STAT5B Y699 in the PR group and an increase in the PR-hGH group, suggesting that the activation of the JAK2-STAT5B signaling pathway occurred after rhGH intervention.

mTOR is a key regulator of cell growth and development and is closely related to liver lipid metabolism and other processes. Using mass spectrometry analysis, we found that the mTOR signaling pathway was activated in the hypophysectomized rats, associated with disturbed lipid homeostasis and oxidative stress. Although there is no clear evidence linking mTOR signals to NADPH regeneration, several studies have shown that mTOR inhibitors can promote G6PD autophagy degradation and exacerbate oxidative stress damage (61); modulate IDH2 expression (62). As mTOR’s role in metabolism is context-dependent and integrates various signals, including nutrient availability, growth factors and energy status, its effects on fatty acid oxidation and NADPH regeneration are probably mediated by complex networks of direct and indirect mechanisms.

**Figure 8:**
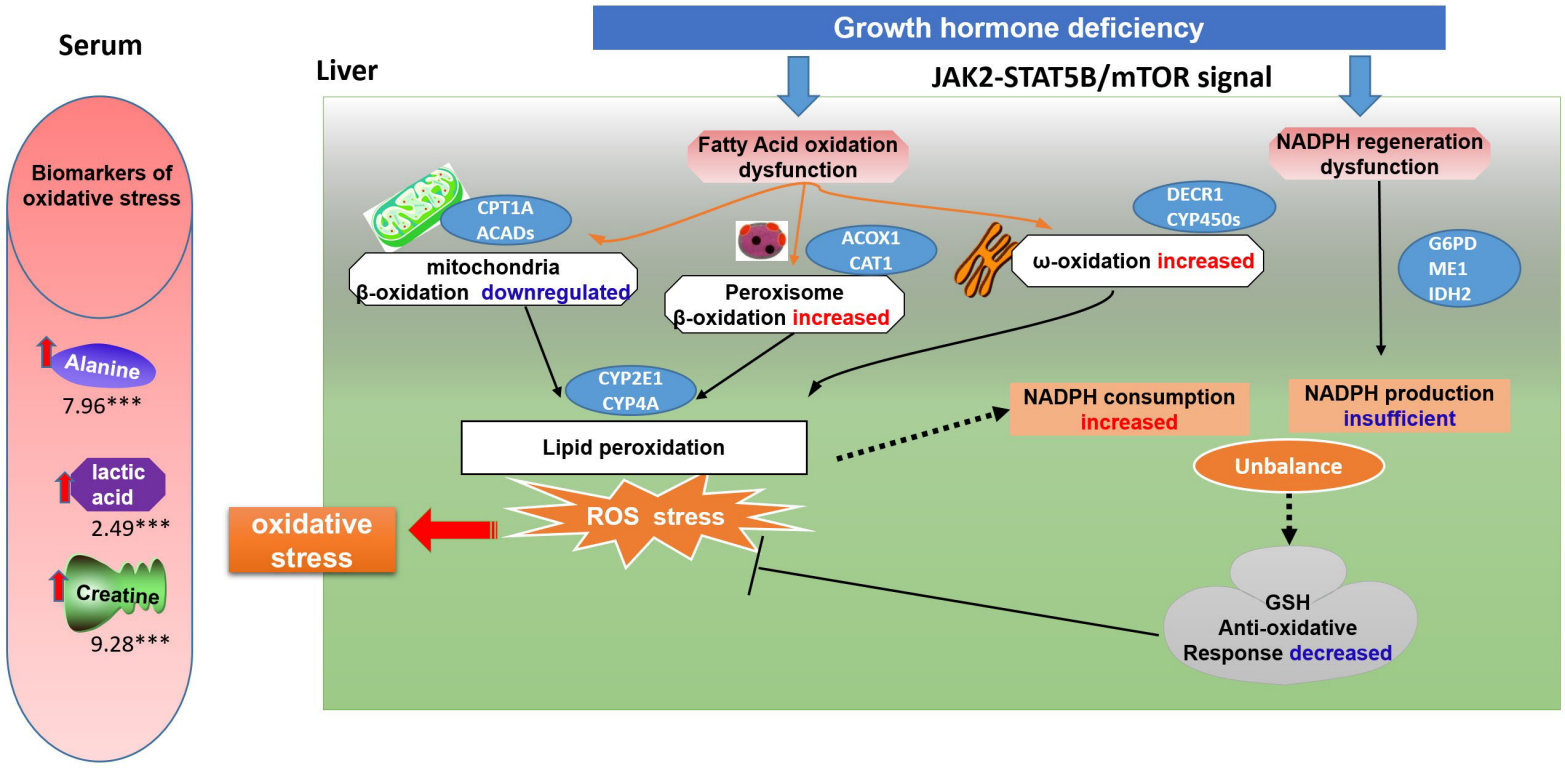
A schematic model of oxidative stress on GH deficiency induced NAFLD. Under the state of GH deficiency, the JAK2-STAT5B, mTOR signaling pathways are disturbed, which may correlate with fatty acid oxidation and NADPH regeneration. To compensate for the insufficient mitochondrial oxidation, peroxisome β-oxidation and ω-oxidation increase, leading to excessive NADPH consumption and lipid peroxidation, resulting in increased ROS. To counteract the detrimental effects of ROS, cells depend on NADPH as a reducing agent to regenerate GSH and maintain cellular redox balance. However, the decreased G6PD activity in PPP and ME1 activity in the malic enzyme pathway result in insufficient NADPH production. The accumulation of oxidative stress and lipid peroxidation due to this contributes to the development of hepatic steatosis and steatohepatitis. Thus, increased oxidative stress biomarkers are present in the serum of individuals with hypopituitarism.

In summary, under the state of GHD, the JAK2-STAT5B, mTOR signaling pathways are disturbed, which may correlate with fatty acid oxidation and NADPH regeneration (**Figure 8**). To compensate for the insufficient mitochondrial oxidation, peroxisome β-oxidation and ω-oxidation increase, leading to excessive NADPH consumption and lipid peroxidation, resulting in increased ROS. To counteract the detrimental effects of ROS, cells depend on NADPH as a reducing agent to regenerate GSH and maintain cellular redox balance. However, the decreased G6PD activity in PPP and ME1 activity in the malic enzyme pathway result in insufficient NADPH production. The accumulation of oxidative stress and lipid peroxidation due to this contributes to the development of hepatic steatosis and steatohepatitis. Thus, increased oxidative stress biomarkers are present in the serum of individuals with HPs. This study provides a correlation between fatty acid oxidation, NADPH regeneration, and the development of NAFLD in the state of GHD. These findings support the hypothesis that antioxidant therapy and mTOR inhibition may mitigate the risk of developing hepatic steatosis in HPs.

## Data availability statement

The data presented in the study are deposited to the ProteomeXchange Consortium (https://proteomecentral.proteomexchange.org) via the iProX partner repository with the dataset identifier PXD052194.

## Ethics statement

The studies involving humans were approved by The Board of Medical Ethics at Ruijin Hospital, Shanghai Jiao Tong University School of Medicine, China. The studies were conducted in accordance with the local legislation and institutional requirements. The participants provided their written informed consent to participate in this study. The animal study was approved by The Board of Medical Ethics at Ruijin Hospital, Shanghai Jiao Tong University School of Medicine, China. The study was conducted in accordance with the local legislation and institutional requirements.

## Author contributions

YZ: Data curation, Funding acquisition, Investigation, Resources, Validation, Writing – original draft, Writing – review & editing. PC: Funding acquisition, Investigation, Supervision, Validation, Writing – review & editing. XF: Data curation, Formal analysis, Funding acquisition, Investigation, Methodology, Software, Validation, Writing – original draft, Writing – review & editing.
