# Supplementary material for: Proteomic and metabolomic analysis of GH deficiency-induced NAFLD in hypopituitarism: insights into oxidative stress
Source: Front Endocrinol (Lausanne). 2024 May 21;15:1371444. doi: 10.3389/fendo.2024.1371444 (PMC11148278; doi:10.3389/fendo.2024.1371444)
Supplement: Supplementary file 1 [file DataSheet_1.docx]

**Supplement Table 1.** Clinical characteristics of the identification study population.

| **Characteristics^a^** | **Congenital hypopituitarism** | **Acquired hypopituitarism** | **Control group** | ***P*-value** |
| --- | --- | --- | --- | --- |
|  | **(n = 83)** | **(n = 51)** | **(n = 90)** |  |
| **Basic information** |  |  |  |  |
| Gender (male/female) | 83/0 | 51/0 | 90/0 | — |
| Age (year) | 25.42 ± 5.79 | 22.88 ± 6.04 | 22.87 ± 2.13 | 0.061 |
| Height (cm) | 166.41 ± 8.51 | 169.61 ± 11.65 | 167.59 ± 5.47 | 0.102 |
| Weight (kg) | 65.88 ± 12.88 | 72.33 ± 19.50 | 66.20 ± 10.97 | 0.008 |
| BMI (Kg/m^2^) | 23.69 ± 3.64 | 24.81 ± 4.74 | 23.53 ± 4.64 | 0.057 |
| **Pituitary hormone deficiency** | | | | |
| GH deficiency | 100% (82/82) | 98.0% (50/51) | — | — |
| LH/FSH deficiency | 97.6% (80/82) | 96.1% (49/51) | — | — |
| TSH deficiency | 91.5% (75/82) | 96.1% (49/51) | — | — |
| ACTH deficiency | 90.2% (74/82) | 94.1% (48/51) | — | — |

^a^The data are the mean ± SD or median (quartile 1–3) for continuous variables and n (%) for categorical variables. Abbreviations: BMI, body mass index; GH, growth hormone; LH, luteinizing hormone; FSH, follicle-stimulating hormone; TSH, thyrotropin; ACTH, adrenocorticotropic hormone.

**Supplement Table 2.** Differential metabolites between hypopituitarism and controls.

| **Number** | **Name** | **Description** | **VIP** | **Fold change** | ***p*-value** |
| --- | --- | --- | --- | --- | --- |
| 1 | M125T92 | 3-Methylhistamine | 1.23 | 0.72 | 0.000 |
| 2 | M118T303 | Betaine | 1.48 | 1.38 | 0.000 |
| 3 | M132T350_2 | Creatine | 5.95 | 9.28 | 0.000 |
| 4 | M114T170 | Creatinine | 9.68 | 0.74 | 0.000 |
| 5 | M188T197 | DL-Indole-3-lactic acid | 1.43 | 2.50 | 0.000 |
| 6 | M89T227_2 | DL-lactate | 6.87 | 1.33 | 0.000 |
| 7 | M149T119 | D-Lyxose | 1.27 | 0.73 | 0.000 |
| 8 | M157T298 | D-Proline | 1.12 | 0.42 | 0.000 |
| 9 | M90T350 | L-Alanine | 1.57 | 7.97 | 0.000 |
| 10 | M175T505_2 | L-Arginine | 4.30 | 1.29 | 0.000 |
| 11 | M162T355_2 | L-Carnitine | 5.56 | 1.12 | 0.000 |
| 12 | M146T399 | L-Glutamate | 2.12 | 1.96 | 0.000 |
| 13 | M188T352 | L-Glutamine | 1.13 | 0.39 | 0.000 |
| 14 | M154T399 | L-Histidine | 1.52 | 1.39 | 0.000 |
| 15 | M130T309 | L-Leucine | 1.01 | 1.15 | 0.000 |
| 16 | M188T502 | L-Lysine | 4.15 | 0.34 | 0.000 |
| 17 | M173T245 | L-Norleucine | 1.31 | 0.33 | 0.000 |
| 18 | M164T255_2 | L-Phenylalanine | 3.60 | 1.43 | 0.000 |
| 19 | M116T315 | L-Proline | 1.63 | 1.47 | 0.000 |
| 20 | M128T300_2 | L-Pyroglutamic acid | 4.02 | 1.28 | 0.000 |
| 21 | M203T256 | L-Tryptophan | 2.18 | 1.52 | 0.000 |
| 22 | M116T298 | L-Valine | 4.04 | 1.57 | 0.000 |
| 23 | M213T168 | m-Chlorohippuric acid | 1.20 | 0.80 | 0.000 |
| 24 | M203T483 | NG,NG-dimethyl-L-arginine(ADMA) | 1.34 | 1.20 | 0.024 |
| 25 | M174T496 | Ornithine | 1.79 | 0.38 | 0.000 |
| 26 | M124T294_2 | Taurine | 4.55 | 1.24 | 0.000 |
| 27 | M212T32 | Indoxyl sulfate | 6.10 | 1.28 | 0.024 |
| 28 | M391T151 | Chenodeoxycholate | 2.74 | 2.54 | 0.000 |
| 29 | M448T210 | Glycochenodeoxycholate | 1.41 | 1.46 | 0.027 |
| 30 | M160T385 | Cyclohexylamine | 1.49 | 1.28 | 0.009 |
| 31 | M319T54 | (+-)12-HETE | 1.88 | 2.29 | 0.000 |
| 32 | M146T378_2 | (3-Carboxypropyl) trimethylammonium cation | 2.15 | 0.83 | 0.000 |
| 33 | M319T36 | 12(R)-HETE | 1.01 | 2.03 | 0.000 |
| 34 | M295T49 | 13(S)-HODE | 1.17 | 1.26 | 0.000 |
| 35 | M313T35_3 | 1-Palmitoylglycerol | 1.06 | 1.16 | 0.000 |
| 36 | M117T162 | 2-Hydroxy-3-methylbutyric acid | 1.38 | 1.22 | 0.013 |
| 37 | M204T308 | Acetylcarnitine | 2.67 | 0.57 | 0.000 |
| 38 | M277T48 | all cis-(6,9,12)-Linolenic acid | 2.46 | 1.22 | 0.008 |
| 39 | M311T36 | Arachidic acid | 1.32 | 0.54 | 0.000 |
| 40 | M187T346 | Azelaic acid | 3.07 | 2.61 | 0.000 |
| 41 | M103T235 | D(-)-beta-hydroxy butyric acid | 1.37 | 0.38 | 0.000 |
| 42 | M316T188 | Decanoyl-L-carnitine | 4.90 | 0.15 | 0.000 |
| 43 | M337T32 | MG(18:2(9Z,12Z)/0:0/0:0)[rac] | 1.52 | 1.70 | 0.000 |
| 44 | M209T35 | Myristoleic acid | 1.23 | 2.84 | 0.000 |
| 45 | M281T101_2 | Oleic acid | 1.49 | 1.10 | 0.029 |
| 46 | M255T48 | Palmitic acid | 7.28 | 0.85 | 0.000 |
| 47 | M201T329 | Sebacic acid | 1.39 | 1.47 | 0.012 |
| 48 | M809T90 | 1,2-dioleoyl-sn-glycero-3-phosphatidylcholine | 2.14 | 1.34 | 0.004 |
| 49 | M468T195 | 1-Myristoyl-sn-glycero-3-phosphocholine | 5.03 | 2.66 | 0.000 |
| 50 | M457T244 | 1-Oleoyl-L-.alpha.-lysophosphatidic acid | 1.18 | 1.61 | 0.000 |
| 51 | M522T188_2 | 1-Oleoyl-sn-glycero-3-phosphocholine | 5.20 | 1.09 | 0.001 |
| 52 | M454T198 | 1-Palmitoyl-2-hydroxy-sn-glycero-3-phosphoethanolamine | 1.40 | 1.80 | 0.000 |
| 53 | M496T191_3 | 1-Palmitoyl-sn-glycero-3-phosphocholine | 28.91 | 1.31 | 0.000 |
| 54 | M628T193 | 1-Stearoyl-2-arachidonoyl-sn-glycerol | 2.87 | 1.61 | 0.000 |
| 55 | M524T187_3 | 1-Stearoyl-2-hydroxy-sn-glycero-3-phosphocholine | 20.89 | 1.35 | 0.000 |
| 56 | M788T146 | 1-Stearoyl-2-oleoyl-sn-glycerol 3-phosphocholine (SOPC) | 4.93 | 1.14 | 0.032 |
| 57 | M70T315 | Diethanolamine | 1.39 | 1.43 | 0.000 |
| 58 | M521T35 | N-Palmitoylsphingosine | 1.22 | 1.60 | 0.000 |
| 59 | M757T58_1 | PC(16:0/16:0) | 1.84 | 1.37 | 0.002 |
| 60 | M169T56 | 3-Hydroxycapric acid | 1.62 | 0.63 | 0.000 |
| 61 | M141T342_2 | 2-Oxoadipic acid | 16.87 | 1.09 | 0.000 |
| 62 | M165T154 | Dihydrothymine | 1.99 | 0.76 | 0.000 |
| 63 | M267T216 | Inosine | 1.04 | 0.16 | 0.000 |
| 64 | M243T162 | Uridine | 1.15 | 0.84 | 0.000 |
| 65 | M120T260_2 | Tyramine | 2.16 | 1.14 | 0.011 |
| 66 | M137T290 | 1-Methylnicotinamide | 1.05 | 2.38 | 0.000 |
| 67 | M129T39 | ketoisocaproic acid | 3.92 | 0.76 | 0.006 |
| 68 | M401T34_3 | 7-Oxocholesterol | 1.49 | 3.42 | 0.000 |
| 69 | M271T29 | Androstanedione | 2.48 | 0.08 | 0.000 |
| 70 | M411T33 | hydropregnenolone sulfate | 2.45 | 0.21 | 0.000 |
| 71 | M395T82 | pregnenolone sulfate | 0.97 | 0.35 | 0.000 |
| 72 | M165T79 | 3-(2-Hydroxyphenyl)propionic acid | 1.42 | 4.18 | 0.004 |


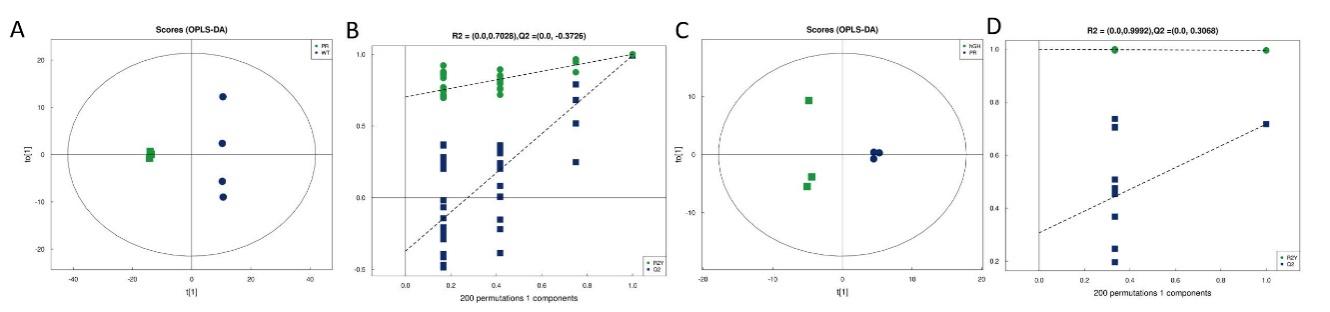


**Supplement Figure 1.** OPLS-DA plot of PR and WT group (A, B), PR-hGH and PR group (C, D).


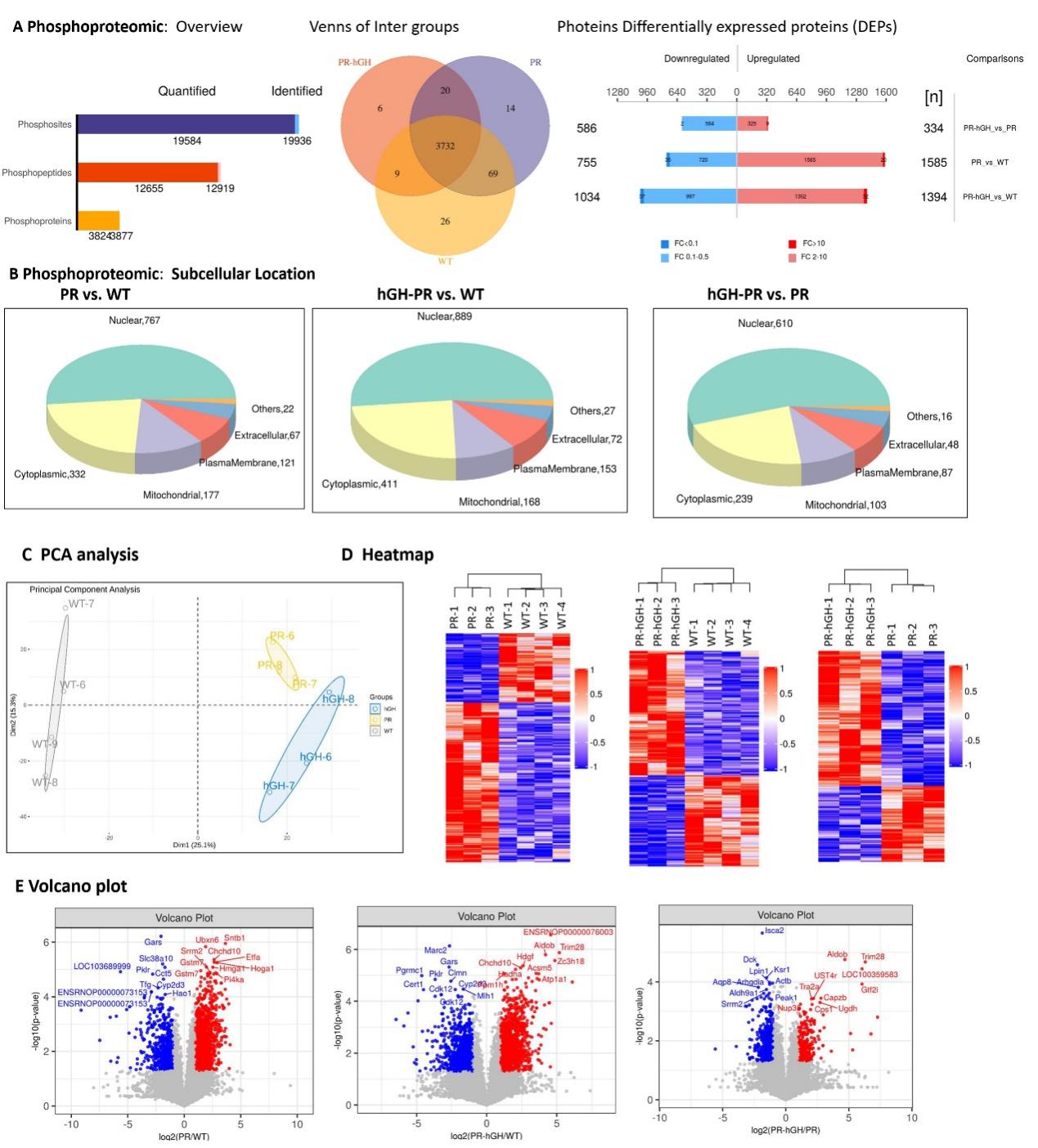


**Supplement Figure 2. Phosphor-Proteome differences between liver samples from PR, PR-hGH and WT.** A: overview of differentially abundant phosphor-proteins; B: Subcellular localization of the differentially abundant proteins; C: Principal component analysis (PCA) clearly separates PR, PR-hGH and WT; D: Heat map of the DEEPs. E: Volcano plot of log10 fold changes. Top10 differentially abundant phosphor-proteins are highlighted.


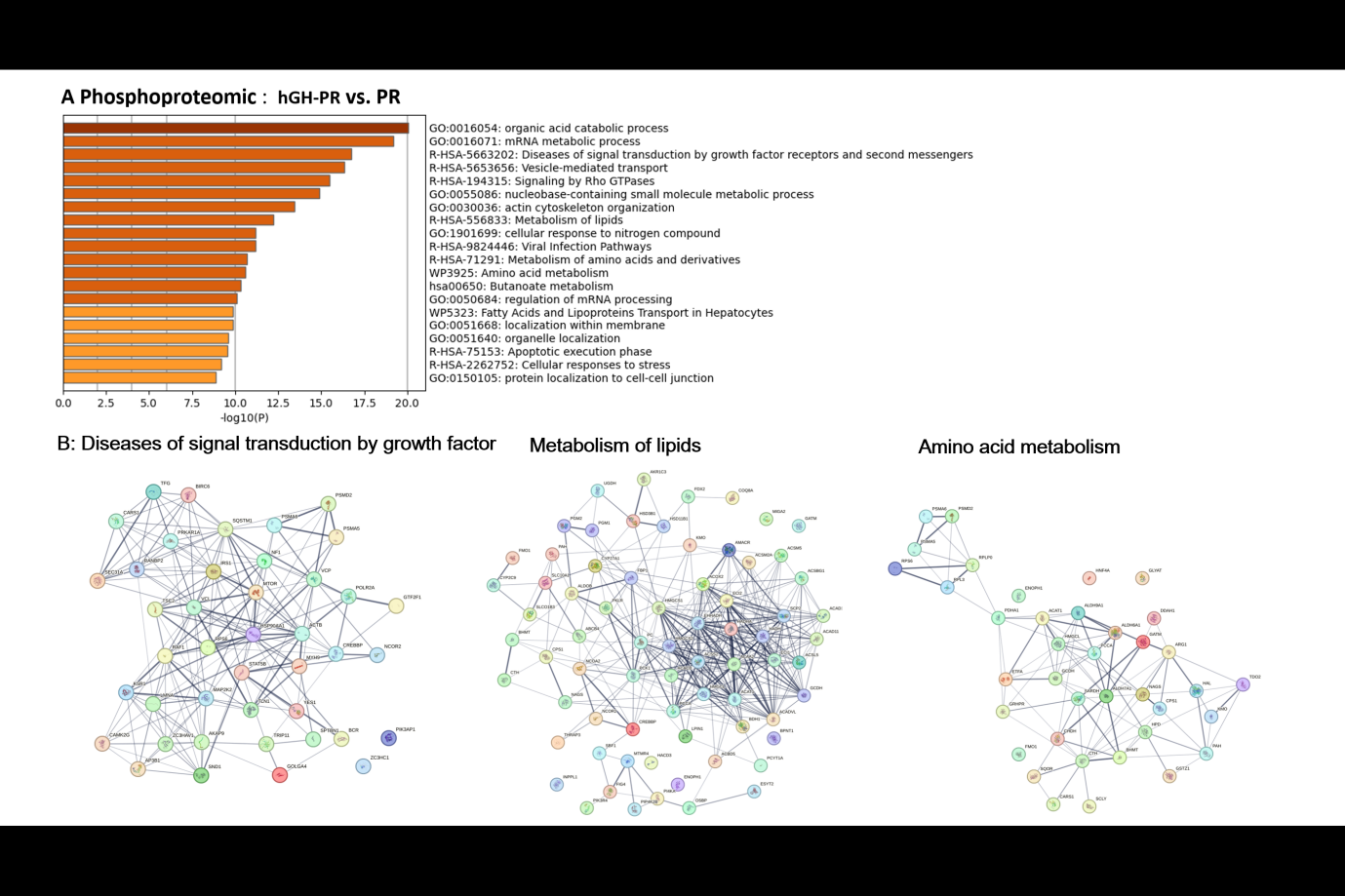


**Supplement Figure 3. Functional enrichment of DEEPs between PR-hGH and PR.** A: ClueGO functional enrichment analysis for phosphor-proteins significantly dysregulated; B: Protein-protein interactions among differentially abundant proteins, the analysis used the term GO Biological Process and KEGG pathways.
